# Urinary Concentrations of Bisphenol Mixtures during Pregnancy and Birth Outcomes: The MAKE Study

**DOI:** 10.3390/ijerph181910098

**Published:** 2021-09-26

**Authors:** Seyoung Kim, Eunjung Park, Eun-Kyo Park, Seulbi Lee, Jeoung-A Kwon, Bo-Hye Shin, Sora Kang, Eun-Young Park, Byungmi Kim

**Affiliations:** 1 National Cancer Control Institute, National Cancer Center, Goyang 10408, Korea; seyoungkim@ncc.re.kr (S.K.); bohye.shin@ncc.re.kr (B.-H.S.); goajoa@ncc.re.kr (E.-Y.P.); 2 Hanyang University Institute for Rheumatology Research, Seoul 04763, Korea; eunjungsays@gmail.com; 3 Department of Occupational and Environmental Medicine, Ewha Medical Research Institute, Seoul 07804, Korea; ekdog1112@naver.com; 4 Department of Epidemiology, University of Michigan School of Public Health, Ann Arbor, MI 48109, USA; Seulbil@umich.edu; 5 Institute of Health Services Research, Yonsei University, Seoul 03722, Korea; kwon.jeounga@gmail.com; 6 Department of Population Health Sciences, Faculty of Life Sciences & Medicine, King’s College London, London WC2R 2LS, UK; 7 Department of Medical Informatics, School of Medicine, Ajou University, Suwon 16499, Korea; kangsora39@gmail.com

**Keywords:** bisphenol, birth weight, birth outcome, mixtures analyses, Bayesian kernel machine regression

## Abstract

Bisphenols are endocrine disruptors that may be associated with altered fetal growth in humans, and they have similar biological functions to mimic hormones. In addition, aggregated chemicals showed an adverse effect although individual concentration was at a low level. However, most studies between bisphenols and birth outcomes have focused on the effect of individual bisphenol. Thus, we explored the associations of urinary bisphenol mixtures with birth outcomes. We conducted a prospective birth cohort study in South Korea. One hundred eighty mother-infant pairs were recruited from 2017 to 2019. Bisphenol A (BPA), bisphenol F (BPF), and bisphenol S (BPS) in one spot urine were analyzed using ultra-performance liquid chromatography–tandem mass spectrometry. We used two statistical approaches to examine potential associations of BPA, BPF, and BPS with birth weight and gestational age: (1) multivariable linear regression; (2) Bayesian kernel machine regression (BKMR). The geometric means of BPA, BPF, and BPS were 2.1, 0.2, and 0.1 μg/L, respectively. In stratified linear analyses by each median value, a higher BPF was positively associated with birth weight (g) (β = 125.5; 95% CI: 45.0 to 205.9). Mixture analyses using BKMR suggested an inverse association between bisphenol mixtures and birth weight. Our findings suggest that in utero bisphenol exposure may influence birth weight and that such relationships may differ considering non-linearity and the combined effect.

## 1. Introduction

Bisphenols are a class of endocrine-disrupting chemicals (EDCs) frequently used in manufacturing polycarbonate plastics and epoxy resins, which are found in various consumer products, such as plastic baby bottles, toys, and epoxy food-can linings [1,2]. Despite their relatively short elimination half-life, bisphenols are associated with adverse health outcomes, such as altered reproduction and neurodevelopment, obesity, and metabolic disorders, among other developmental and chronic impairments [3]. The increased use of bisphenol A (BPA) led scientists, regulators, and the general public to voice concerns over its safety. Since 2010, several governments such as Canada, the European Union, US Food Drug Administration (FDA), and South Korea have banned the use of BPA in baby bottles and sippy cups [4]. Bisphenol F (BPF) and bisphenol S (BPS) have been widely used as substitutes in manufacturing various BPA-free products [1,5]. Products labeled ‘BPA free’ typically contain BPS [5].

Bisphenols influence on physiological receptors in different pathways [6]. BPA can pass the placenta, so fetuses are directly exposed to BPA [7,8,9]. Exposure to BPA during gestation may affect fetal growth through multiple hormone-mediated mechanisms by mimicking estrogen, inhibiting androgen production, altering thyroid signaling, and causing oxidative stress [8,9,10]. Several human studies have described a positive association between BPA exposure during pregnancy and birth growth [11,12,13], whereas others have suggested no association [3,14,15] or a negative association [1,8,16]. However, the impact of prenatal exposure to BPA on birth growth remains unclear due to inconsistent findings from epidemiological studies [2].

Considering the toxicity of BPA and consumer concerns, manufacturers have been banned from using BPA in their products, with gradual transitions to using bisphenol analogs, such as BPF and BPS. Thus, BPF and BPS have been widely used in manufacturing BPA-free products [14,15]. Some recent studies showed that urinary BPS levels were significantly associated with low birth weight [16] and small for gestational age [17], while others found no association between urinary BPS levels and birth size [18,19]. Maternal BPF exposure also was associated with smaller birth size in some studies [8,17], but no association was found in others [16].

Although these previous studies found that exposure to environmental chemicals during pregnancy may be associated with adverse fetal growth, most did not consider the aggregate effect of environmental chemical exposure on infant health [20]. Among the few studies that did quantify the impact of exposure to multiple bisphenols on fetal growth, most suggested that aggregate exposure to multiple bisphenols may be associated with adverse fetal development, but they had a limitation by examining only specific classes of bisphenols. To obtain a deeper understanding of the potential effects between exposure to multiple types of bisphenols and fetal development, we used two different methods to estimate the association between bisphenol mixtures and birth outcomes among mother-child pairs in the Mother and Kids Environmental Health (MAKE) study.

## 2. Materials and Methods

### 2.1. Study Design and Participants

The MAKE study was a community-based prospective birth cohort study in South Korea to examine the effects of maternal chemical exposure during pregnancy on fetal growth. Pregnant Korean women aged at least 18 years were eligible to enroll. The details of the MAKE study have been described previously [5]. Briefly, from January 2017 to August 2020, pregnant women were recruited from the metropolitan region near Seoul, South Korea. In total, 261 participants who provided their informed consent completed the questionnaires and health examination. In the present study, 244 pregnant women who provided their infant birth information were included. Among them, we excluded women with multiple births (*n* = 8) and no available urinary bisphenol concentration data (*n* = 56). Therefore, 180 pregnant women were included in the final analysis. This study was approved by the Institutional Review Boards of National Cancer Center, Korea.

### 2.2. Sample Collection and Measurement of Urinary Bisphenol Concentrations

Spot urine samples were collected from each participant while visiting the center and then were frozen at −70 °C. To measure the urinary bisphenol concentrations, a 1-mL sample was transferred to a glass test tube, and then internal standards of 50 μg/L, 1 mL of 2 M sodium acetate, and 50 μg/L of β-glucuronidase (*Escherichia coli* K12; Roche Biomedical, Mannheim, Germany) were added. The samples were enzymatically hydrolyzed for 16 h in a constant temperature bath at 37 °C and then cooled down by adding 100 μg/L of 2 N HCl. After solid phase extraction to remove impurities, the samples were eluted with methanol and then analyzed using ultra-performance liquid chromatography–tandem mass spectrometry (Agilent 6490; Santa Clara, CA, USA).

Internal quality assurance and control (QC) were performed such as linearity, accuracy, precision, and method detection limits. For quality control, seven samples with a concentrated standard 3 to 10 times the detection limit were measured with the same procedures mentioned above. The limit of detection (LOD) was calculated by multiplying the standard deviation of the seven replicates by 3.14 (98% freedom, six degrees of freedom). The precision and accuracy measurements were calculated by multiplying by the relative standard deviation of more than five replicated measurements. The acceptable range of precision and accuracy was 85–115%. The correlation coefficient of the calibration curve was more than 0.999. The LOD for BPA, BPF, and BPS were 0.071, 0.083, and 0.020 μg/L, respectively.

For external QC, each analytical laboratory regularly participated in international and national quality control programs. Internationally, all the laboratories participated in the 61st to 63rd programs of the German External Quality Assessment Scheme (G-EQUAS) and also participated in the domestic quality assurance program by the National Institute of Environmental Research (NIER) in South Korea. All the analytical results of the laboratories were qualified within the tolerance ranges by the managers of G-EQUAS and NIER.

### 2.3. Assessment of Birth Outcomes

After delivery, we obtained birth information, including the birth weight (g) and gestational age (week) from medical record. Gestational age at delivery was estimated from the first date of the last menstrual cycle or the first date of ultrasonographic estimation if the date of the last menstrual period was unreliable or if a significant difference occurred between the last menstrual cycle date and ultrasonographic estimation date (>10 days).

### 2.4. Statistical Analysis

Urinary bisphenol concentrations below the LOD were assigned to LOD/√2 [21], and all the urinary bisphenol concentrations were log_10_-transformed for normality assumption. The urinary bisphenols were corrected for specific gravity (SG) and measured using a urine analyzer (Urisys 2400, Roche, Mannheim, Germany) to adjust the urinary dilution [22]. The SG-correction formula was as follows:

$$\mathrm{SG}-\mathrm{corrected}\;\mathrm{concentration}\;(\mu g/L)=\mathrm{Bisphenol}\;\mathrm{concentration}\times \frac{\left({\mathrm{SG}}_{p}-1\right)}{\left({\mathrm{SG}}_{i}-1\right)}$$

where ${\mathrm{SG}}_{p}$ is the study population median SG (1.018) and ${\mathrm{SG}}_{i}$ is the individual SG.

We used Student’s *t*-test to assess the differences in the birth weight and gestational age according to the maternal and infant characteristics. We selected potential confounders, known as influential factors, for bisphenol exposure and birth outcomes from previous studies with a directed acyclic graph (Figure S1). All the multivariable-adjusted models included the following confounders: maternal age (<30 or ≥30 years), educational level (<university or ≥university), household income per month (<3000 or ≥3000 Korean won), smoking status (yes or no), drinking status (yes or no), exercise (yes or no), body mass index (BMI, <23 or ≥23 kg/m^2^) [23] before pregnancy, parity (nulliparous or multiparous), infant sex (male or female), and gestational age (weeks).

#### 2.4.1. Linear Analyses

To estimate the linear associations between each urinary bisphenol and birth outcomes, we used multivariable linear regression models using the SAS 9.4 software (SAS Institute Inc., Cary, NC, USA). We used three models: unadjusted model, multivariable-adjusted model, and mutually adjusted model.

#### 2.4.2. Mixture Analyses

We conducted further analyses using Bayesian kernel machine regression (BKMR) [24], with the BKMR R package [25]. BKMR can estimate the health effect of environmental exposures considering the nonlinear and nonadditive effects of exposures using a multivariable exposure-response function. Computational and theoretical explanations of BKMR have been described elsewhere [24,25].

BKMR with a gaussian kernel function is used to estimate the individual and overall joint association of bisphenols with birth outcomes, and the formula is given by:

$$Y_{i}=h\left(C_{BPA},C_{BPF},C_{BPS}\right)+x_{i}^{\prime }\beta +e_{i}$$

where $Y_{i}$ denotes the birth outcomes for an individual ***i*** (***i*** = 1, …, *n*), ***C*** is the concentrations of bisphenols, ***h***() denotes the unknown exposure-response function to be estimated, $x_{i}$ is a vector of the confounders, β represents the effect of the confounders, and $e_{i}$ indicates residuals from normal distribution. Additionally, we estimated posterior inclusion probabilities (PIPs) for each bisphenol using component-wise variable selection. PIPs represent the relative importance of each variable and range from 0 to 1, with values closer to 1 indicating a substantial exposure to the outcomes [25].

As BKMR calculates the high-dimensional estimation of the exposure-response surface, it can visualize these associations by defining the cross-sectional surface. Thus, we present the following in this study: (1) the univariate exposure–response relationship of one bisphenol with birth outcomes, considering that others are fixed at their median value and all the covariates have a constant value; (2) the association of one bisphenol increases by a certain percentile (i.e., 10th to 50th percentile) with birth outcomes at various quantile values of others, considering that all the covariates have a constant value; (3) the bivariate exposure–response relationship of one bisphenol with birth outcomes at various quantile values of another bisphenol, considering that the other bisphenol is fixed at its median value and all the covariates have a constant value; (4) the overall joint association of all bisphenols increase by five percentiles with birth outcomes as referenced to their 50th percentile, considering that all the covariates have a constant value.

The BPA, BPF, and BPS concentrations were scaled to log_10_-transformed values and centered to handle skewed data, and all the BKMR models were adjusted for potential confounders. BKMR was implemented with the R package *bkmr* using R software (version 4.0.2, R Core Team 2020, Vienna, Austria).

## 3. Results

### 3.1. Maternal and Infant Characteristics and the Birth Outcomes

Table 1 shows the characteristics of the study participants and birth weight and gestational age according to these characteristics. Most participants were ≥30 years at enrollment, received a high education level, and their household income was higher than the domestic average in South Korea. Regarding lifestyle, drinkers and smokers comprised 21.7% and 11.1% of the participants, respectively. Most of the pregnant women had a low BMI (<23) before pregnancy. The proportion of the male and female infants was similar, and most were first born. Preterm birth infants (<37 gestational weeks) represented 3.9% of the total, and low birth weight infants also represented 3.9% of the total. Among the characteristics, the factors affecting the birth weight were the infant’s sex and gestational weeks, and the factor showing a significant difference in gestational age was infant low birth weight.

### 3.2. Urinary Bisphenol Concentrations

The detection rates of bisphenols above the LOD in urine samples followed the order of BPA (96.2%), BPF (84.4%), and BPS (66.1%). The SG-corrected geometric means (GM) of the BPA, BPF, and BPS concentrations were 2.1, 0.2, and 0.1 μg/L, respectively (Table 2). The maternal BPA, BPF, and BPS concentrations were positively correlated (ρ from 0.10 to 0.22) (Figure S2).

### 3.3. Multivariable Linear Regression on Birth Outcomes

We analyzed the associations of bisphenols with birth weight and gestational age using linear regression models (Table 3). After adjusting for potential confounders and other bisphenols, BPA and BPF showed a positive trend, respectively, for the birth weight (BPA: β = 5.5, 95% confidence interval [CI]: −43.6 to 54.7, *p*-value = 0.82; BPF: β = 38.5, 95% CI: −18.1 to 95.1, *p*-value = 0.18) and gestational age (β = 0.05, 95% CI: −0.16 to 0.27, *p*-value = 0.61 of BPA; β = 0.13, 95% CI: −0.12 to 0.38, *p*-value = 0.29 of BPF). However, BPS was negatively associated with the birth weight (β = −44.2, 95% CI: −92.7 to 4.4, *p*-value = 0.07) and gestational age (β = −0.09, 95% CI: −0.30 to 0.13, *p*-value = 0.43); additionally, all the linear associations were not statistically significant. However, after stratified analyses by the median concentration of each bisphenol, a higher BPF level was significantly associated with increased birth weight (β = 125.5; 95% CI: 45.0 to 205.9; *p*-value = 0.003) (Table 4).

### 3.4. BKMR on the Birth Outcomes

**Figure 1:**
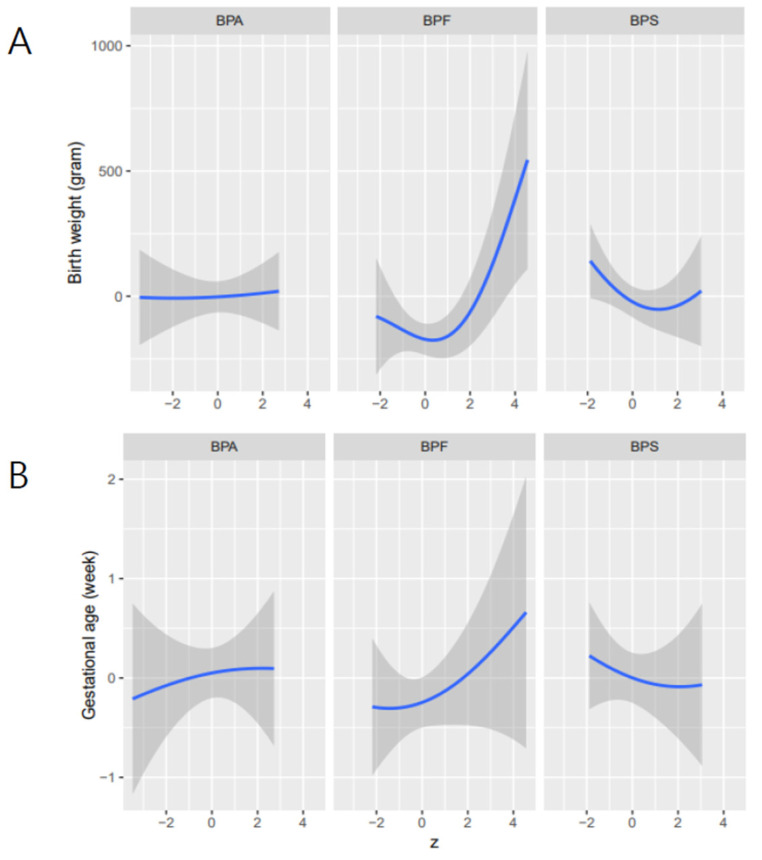
Univariate exposure–response functions and 95% credible intervals for each bisphenol (BPA, BPF, BPS) and the birth weight (**A**) and gestational age (**B**) when the other bisphenols are fixed at their medians, respectively. These results were estimated by Bayesian kernel machine regression. Models were adjusted for maternal age, education, family income, smoking status, drinking status, body mass index, exercise, parity, infant sex, and gestational weeks (only in the case of birth weight).

We conducted BKMR using component-wise variable selection to examine the potential nonlinear and interactive effects and overall effect of bisphenols on the birth outcomes, adjusting for potential confounders. The estimated PIPs on the birth weight for BPA, BPF, and BPS were 0.11, 0.78, and 0.48, respectively, and the PIPs of the gestational age for BPA, BPF, and BPS were 0.21, 0.31, 0.25, respectively. Figure 1 shows the exposure-response functions and 95% credible intervals for each bisphenol and birth weight and gestational age, considering that other bisphenols are fixed at their medians values and potential confounders are held constant.

**Figure 2:**
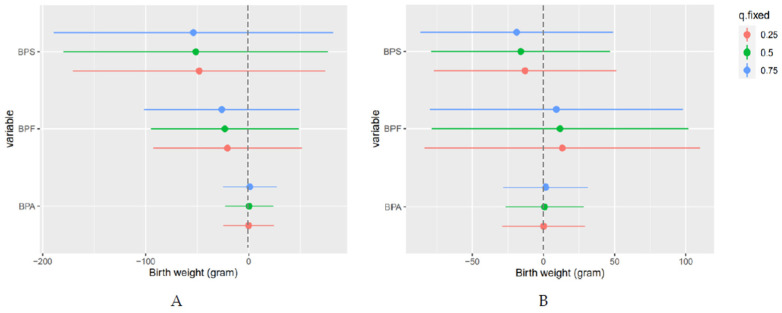
Change in average birth weight (estimates and 95% credible intervals, gray dashed line at the null) (**A**) for increase in each bisphenol from its 10th to 50th and (**B**) for increase in each bisphenol from its 50th 90th when the other bisphenols were set at their median values. Models were adjusted for maternal age, education, family income, smoking status, drinking status, body mass index, exercise, parity, infant sex, and gestational weeks (only in the case of birth weight).

In this study, we found nonlinear associations of BPF and BPS on birth weight. For BPF, an increase in BPF from the 10th to 50th percentile (0.1 to 0.2 μg/L; Table 2) was associated with a 23.2 g (SD = 36.6) decrease in the average birth weight, whereas an increase in BPF from 50th to 90th percentile (0.2 to 0.8 μg/L; Table 2) was associated with a 11.6-g (SD = 46.0) increase in the average birth weight (Figure 2). Regarding BPS, an increase in BPS from the 10th to 50th percentile (0.01 to 0.05 μg/L; Table 2) was associated with a 51.3-g (SD = 65.5) decrease in the average birth weight, whereas an increase in BPF from the 50th to 90th percentile (0.05 to 0.2 μg/L; Table 2) was associated with a 16.0-g (SD = 32.1) decrease in the average birth weight (Figure 2). However, no change in the birth weight was observed when the BPA concentration increased, showing a flattening dose–response curve. In gestational age, we observed an upward for BPA and BPF and downward for BPS. However, their dose–response curve had wide credible intervals because of the small number of participants represented.

To examine potential pairwise interactions among the bisphenols, particularly due to the nonlinearity for BPF and BPS, we estimated the predicted change in birth weight and gestational age, considering the other bisphenol patterns at 25th, 50th, or 75th percentiles. Figure S3 shows possible interactions between BPF and BPS, where the negative slope of lower BPF levels with birth weight is slightly steeper at higher BPS levels than at lower BPS levels and the negative slope of BPS with birth weight is slightly steeper at higher BPF levels than at lower BPF levels. The interactions of bisphenols with gestational weeks were not noticeable in this study population.

**Figure 3:**
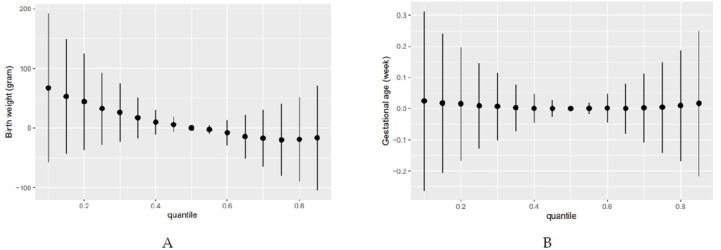
Overall effect of bisphenol mixture (BPA, BPF, BPS) exposure on the birth weight (**A**) and gestational age (**B**) (estimates and 95% credible intervals) by Bayesian kernel machine regression. This plot compares the change in the birth outcomes when all bisphenol exposures are at a particular quantile (*x*-axis) compared to when all bisphenols exposure are at 50th percentile.

Finally, we also examined the overall effect of bisphenol mixtures on birth outcomes. Figure 3 shows the estimated change in the average birth weight and gestational age when all the bisphenols were at a certain percentile (from the 10th to 85th percentile) compared with when they were at their median values (50th percentile). For example, bisphenols set at their 80th percentile were associated with a 20.1-g (SD: 37.1) decrease compared with when all the bisphenols were set at their 50th percentiles. Increasing levels of the bisphenol mixture were associated with a decrease in birth weight. However, we found no changes in gestational age.

## 4. Discussion

In this prospective birth cohort study, we examined the associations of the gestational concentrations of bisphenols with birth weight and gestational age. We found that higher BPF levels were significantly associated with increased birth weight. We observed a non-linear association of BPF and BPS on birth weight and increasing levels of the bisphenol mixture were associated with decreased birth weight. However, we found no changes in gestational age.

In the current study, urinary BPA levels were much higher than those of its analogs. The results of our individual bisphenol analyses were similar to those previously reported in investigations using the MAKE study [5]. Compared with our result (2.1 ng/mL), the BPA level of our participants was higher than those of pregnant women from China (GM: 0.9 ng/mL) [1], Korea (GM: 1.3 ng/mL) [12], and Canada (median: 0.8 ng/mL) [20] but lower than those from Puerto Rico (GM: 2.6 ng/mL) [26], France (median: 3.1 ng/mL) [27], and Spain (GM: 2.3 ng/mL) [28]. The differences in the results could be due to lifestyle, years of sample collection, and different national regulations on BPA. The findings of our study conducted in 2017–2019 were comparable to that of 788 samples of the Korean general population recruited in 2006–2011. Although the samples were different, the BPA exposure in Korea did not decrease significantly compared with that a decade ago. The data concerning the blood exposure levels of BPA analogs in pregnant women are limited currently. The median concentration of BPF (0.2 μg/L) in this study was lower than that in pregnant women from China (0.7 ng/mL) [1] and the Netherlands (0.6 ng/mL) [29]. BPS had the lowest detection rate of the three bisphenols we analyzed; the median BPS concentration (0.1 μg/L) was lower in pregnant women than in those in other countries (China: 0.2 ng/mL, Netherlands: 0.4 ng/mL) [18,29]. BPF and BPS exposure data were rare, particularly for pregnant women and were insufficient for accurate comparison. More human studies on BPA analogs exposure are needed.

Previous studies have found that maternal bisphenol exposure is associated with birth weight. However, the impact of prenatal exposure to BPA on size at birth is unclear because of the inconsistent findings from current epidemiological studies [2]. Our results are consistent with previous studies, which found that maternal BPA exposure is not associated with birth weight. The present study and studies from the United States [30,31], Spain [28], and China [1] did not support the associations of BPA exposure before and during pregnancy with fetal growth. However, a nested case–control study from China observed that prenatal BPA exposure was a risk factor for low birth weight [32]. Similarly, two birth cohort studies reported that higher urinary BPA concentrations during pregnancy were associated with reduced birth weight in the Netherlands [33] and China [34]. Another nested case–control study, from the United States, revealed an association of the urinary BPA concentrations during pregnancy with an increased risk of preterm delivery [35]. By contrast, a study from South Korea [11] and USA [13] reported a positive association of urinary BPA concentrations with birth weight. Therefore, more studies are needed to investigate the effect of exposure to BPA during pregnancy on fetal growth in humans.

Similar to BPA, two birth cohort studies reported that maternal exposure to BPS and BPF was inversely associated with size at birth [1,17], while another reported no significant associations [16]. One study reported that the urinary BPS concentration in the third trimester had the significant association with increased pregnancy duration, but not with reduced birth weight [18]. Another study in the USA suggested negative associations between the urinary BPS concentrations during pregnancy with multiple points, and birth weight in boys [30]. In the present study, only higher BPF levels and birth weight showed a significant positive association, and we observed a J-shaped pattern between BPF and birth weight—a slope change that decreases birth weight at low concentrations but increases at high concentrations (Figure 2). Additional studies of EDCs, including BPA, reported that EDCs had a non-linear dose–response curve on adverse effects in cell, animal, and human studies [36]. Some researchers recommended that nonmonotonicity should be considered as a basis when evaluating chemicals acting similar to hormones [36,37]. However, few assessed nonlinearity and most were conducted using a categorical exposure variable without a statistical method [38]. Thus far, no study has investigated the nonlinearity association between bisphenol and birth outcome considering other bisphenols. Therefore, we suggest the possibility of a non-linear association between BPF and birth weight because nonmonotonicity is a general characteristic of EDCs. In addition, these non-linear exposure–response relationships could affect the inconsistent results in previous studies assuming a linear relationship. Thus, further investigations are warranted in larger prospective birth cohorts considering the non-linear association of EDCs.

Previous studies have assessed the association between individual chemical biomarkers and birth outcomes. Few have examined the effect of aggregate environmental exposures on birth outcomes [39,40,41,42]. To our best knowledge, no epidemiological study has estimated the relationship between the exposure of bisphenols, including BPA, BPS, and BPF, during pregnancy and birth outcome. One study has compared multiple measures of these chemicals during pregnancy in relation to birth size. Characterizing multiple chemical exposures in the HOME study from the USA identified inverse associations of select organochlorine compounds, some phenols, and cadmium with birth length, but not BPA and with other neonatal outcomes [39]. The study included BPA but not BPF and BPS. In our results, bisphenol mixtures including BPA, BPF, and BPS decreased the birth weight while individual bisphenols showed different patterns, such that BPA and BPS showed an inverse association, but BPF showed a positive association, with birth weight. These results suggest that individual chemicals and mixtures may have different effects on the same health outcome. Many EDCs use similar pathways to mimic hormones, such as binding to hormone receptors, leading to competitive binding. Aggregated EDCs showed an adverse effect even though individual substances were at a lower concentrations than the effective concentration [43]. In cell studies, BPA can increase the expression of estrogen receptors, making cells more vulnerable to other EDCs [38,44].

The metabolism of BPS and BPF have not been well studied. However, several experiments have showed that BPF metabolism and distribution are similar to those of BPA [14]. BPA exposure increases the serum insulin levels, decreases adiponectin secretion, resulting in alternate transcriptional modification in adipocytes [5]. An experimental study showed that BPA and its derivatives may induce adipocyte differentiation through the 3-kinase and Akt kinase pathways, which promote lipid accumulation in adipocyte and the expression of genes involved in adipogenesis [45]. BPF can also produce cytotoxicity, cellular dysfunction, DNA damage, and chromosomal abnormalities [14,46] and decreases a production and secretion of adiponectin [47]. In addition, exposure to BPF provokes an estrogenic effect and induces uterine growth in rats [48,49]. In this study, BPF may induce fetal volume growth by BPF accumulated in the fetus, thereby inducing disorders, such as obesity and metabolic syndrome. In addition, as exposure accumulates in the placenta and fetus, BPF functions as an adipogenesis factor in the human body.

Our study has several limitations. First, the specificity of the bisphenol mixture exposure assessment has not been evaluated in other studies. Second, our study sample size was small, which may reduce the statistical power to obtain the sufficient precision of our estimates [50]. Finally, this study was limited by using a spot measurement of urinary bisphenol concentration that may not reflect the dynamics of bisphenol exposure during pregnancy. However, because the exposure value has a short lifespan in the body, multiple measurements would also not reflect exposure precisely. Despite the above limitations, this is the first study to assess the combined effect of bisphenol mixtures, including BPA, BPF, and BPS, considering non-linearity and interactions.

## 5. Conclusions

Higher exposure to BPF is associated with increased birth weight. However, the overall joint association of bisphenol mixture exposure with birth weight suggests an inverse association with birth weight. Thus, individual chemical exposure and mixture exposure may have different effects on the same health outcome. Further studies are required to substantiate these findings considering non-linearity and the combined effect.

## Figures and Tables

**Table 1:** Maternal and infant characteristics of the study population (*n* = 180).

		Birth Weight (g)		Gestational Age (Weeks)	
	N (%)	Mean ± SD ^a^	*p*-Value	Mean ± SD ^a^	*p*-Value
***Maternal Characteristic***					
Age at enrollment (years)					
<30	41 (22.8)	3251 ± 409	0.92	39.21 ± 2.08	0.98
≥30	139 (77.2)	3244 ± 394		39.20 ± 1.29	
Education level					
<University	72 (40)	3191 ± 365	0.13	39.35 ± 1.41	0.30
≥University	108 (60)	3281 ± 414		39.11 ± 1.55	
Household per month (1000 KRW)					
<3000	47 (26.1)	3240 ± 449	0.93	39.15 ± 1.38	0.77
≥3000	133 (73.9)	3247 ± 378		39.23 ± 1.54	
Smoking status					
Yes	20 (11.1)	3198 ± 535	0.67	38.98 ± 1.97	0.57
No	160 (88.9)	3251 ± 377		39.24 ± 1.43	
Drinking status					
Yes	39 (21.7)	3224 ± 411	0.70	38.84 ± 1.98	0.17
No	141 (78.3)	3251 ± 394		39.31 ± 1.33	
Exercise					
Yes	30 (16.7)	3162 ± 365	0.21	39.24 ± 1.07	0.87
No	150 (83.3)	3262 ± 401		39.20 ± 1.57	
BMI at prepregnancy (kg/m^2^)					
<23	134 (74.4)	3236 ± 387	0.59	39.32 ± 1.45	0.09
≥23	46 (25.6)	3272 ± 424		38.88 ± 1.62	
***Infant Characteristic***					
Sex					
Male	91 (50.6)	3335 ± 396	0.0019	39.23 ± 1.39	0.80
Female	89 (49.4)	3154 ± 377		39.18 ± 1.61	
Parity					
Nulliparous	125 (69.4)	3224 ± 386	0.28	39.29 ± 1.60	0.20
Multiparous	55 (30.6)	3294 ± 418		39.01 ± 1.20	
Gestational age (weeks)					
<37	7 (3.9)	2556 ± 368	<0.0001	34.40 ± 2.87	<0.0001
≥37	173 (96.1)	3273 ± 372		39.40 ± 1.03	
Birth weight (g)					<0.0001
<2500	7 (3.9)	2314 ± 229	<0.0001	37.00 ± 2.17	
≥2500	173 (96.1)	3283 ± 353		39.30 ± 1.40	

Abbreviations: BMI, body mass index; KRW, Korean won (in 10,000 Korean won; $US10). ^a^ Mean ± SD: mean ± standard deviation.

**Table 2:** Distribution of the maternal urinary bisphenol concentration of the study population (*n* = 180).

SG Corrected (μg/L)	GM (GSD)	LOD	N (%) > LOD	Percentile					
				10th	25th	50th	75th	90th	Max
BPA	2.1 (2.9)	0.071	173 (96.2)	0.7	1.2	2.1	4.0	7.1	36.8
BPF	0.2 (2.6)	0.083	152 (84.4)	0.1	0.1	0.2	0.4	0.8	18.0
BPS	0.1 (3.0)	0.020	119 (66.1)	0.01	0.03	0.05	0.1	0.2	1.5

Abbreviations: GM, geometric mean; LOD, limit of detection; SG, specific gravity; BPA, bisphenol A; BPF, bisphenol F; BPS, bisphenol S.

**Table 3:** Associations of the bisphenol concentrations with birth weight and gestational age for each 10-fold increase in the maternal urinary bisphenol level (specific-gravity corrected, μg/L).

	BPA		BPF		BPS	
	β [95% CI]	*p*-Value	β [95% CI]	*p*-Value	β [95% CI]	*p*-Value
**Birth Weight**						
Model 1	1.5 (−54.1 to 57.0)	0.96	36.5 (−24.6 to 97.5)	0.24	−38.1 (−91.4 to 15.2)	0.16
Model 2	4.0 (−45.0 to 52.9)	0.87	27.4 (−27.7 to 82.4)	0.33	−36.0 (−83.0 to 11.1)	0.13
Model 3	5.5 (−43.6 to 54.7)	0.82	38.5 (−18.1 to 95.1)	0.18	−44.2 (−92.7 to 4.4)	0.07
**Gestational Age**						
Model 1	0.09 (−0.12 to 0.30)	0.41	0.04 (−0.02 to 0.10)	0.24	−0.07 (−0.27 to 0.13)	0.49
Model 2	0.06 (−0.16 to 0.27)	0.59	0.12 (−0.12 to 0.36)	0.34	−0.05 (−0.26 to 0.15)	0.61
Model 3	0.05 (−0.16 to 0.27)	0.61	0.13 (−0.12 to 0.38)	0.29	−0.09 (−0.30 to 0.13)	0.43

Abbreviations: BPA, bisphenol A; BPF, bisphenol F; BPS, bisphenol S. Note: Model 1 was the unadjusted model. Model 2 was adjusted for maternal age, education, family income, smoking status, drinking status, body mass index, exercise, parity, infant sex, and gestational weeks (only in the case of birth weight). Model 3 was Model 2 + adjusted for the other bisphenols.

**Table 4:** Associations of the bisphenol concentration with birth weight and gestational age stratified by their median concentration.

	BPA		BPF		BPS	
	β [95% CI]	*p*-Value	β [95% CI]	*p*-Value	β [95% CI]	*p*-Value
**Birth Weight**						
**<Median**	62.3 (−33.1 to 157.8)	0.20	92.9 (−128.1 to 313.8)	0.41	−140.7 (−307.9 to 26.4)	0.09
**≥** **Median**	21.8 (−106.0 to 149.9)	0.74	**125.5 (45.0 to 205.9)**	**0.003**	30.8 (−47.7 to 109.3)	0.44
**Gestational Age**						
**<Median**	0.17 (−0.25 to 0.59)	0.43	−0.27 (−1.28 to 0.73)	0.59	−0.26 (−0.85 to 0.34)	0.40
**≥** **Median**	0.13 (−0.46 to 0.72	0.66	0.15 (−0.19 to 0.49)	0.38	0.01 (−0.45 to 0.47)	0.97

Note: The models were adjusted for maternal age, education, family income, smoking status, drinking status, body mass index, exercise, parity, infant sex, gestational weeks (only in the case of birth weight), and the other bisphenols.

## Data Availability

The data presented in this study are available on request from the corresponding author.

## Supplementary Materials

The following are available online at https://www.mdpi.com/article/10.3390/ijerph181910098/s1, Figure S1: Directed acyclic graph (DAG). Figure S2: Correlation of BPA, BPF, and BPS. Figure S3: Bivariate exposure-response functions for one bisphenol (expos1) at quantiles of another bisphenol (expos2) on the (a) birth weight and (b) gestational age.
