# Supplementary material for: Urinary Concentrations of Bisphenol Mixtures during Pregnancy and Birth Outcomes: The MAKE Study
Source: Int J Environ Res Public Health. 2021 Sep 26;18(19):10098. doi: 10.3390/ijerph181910098 (PMC8508042; doi:10.3390/ijerph181910098)
Supplement: Supplementary file 1 [file ijerph-18-10098-s001.zip › ijerph-1366917-Supplementary Materials.pdf]

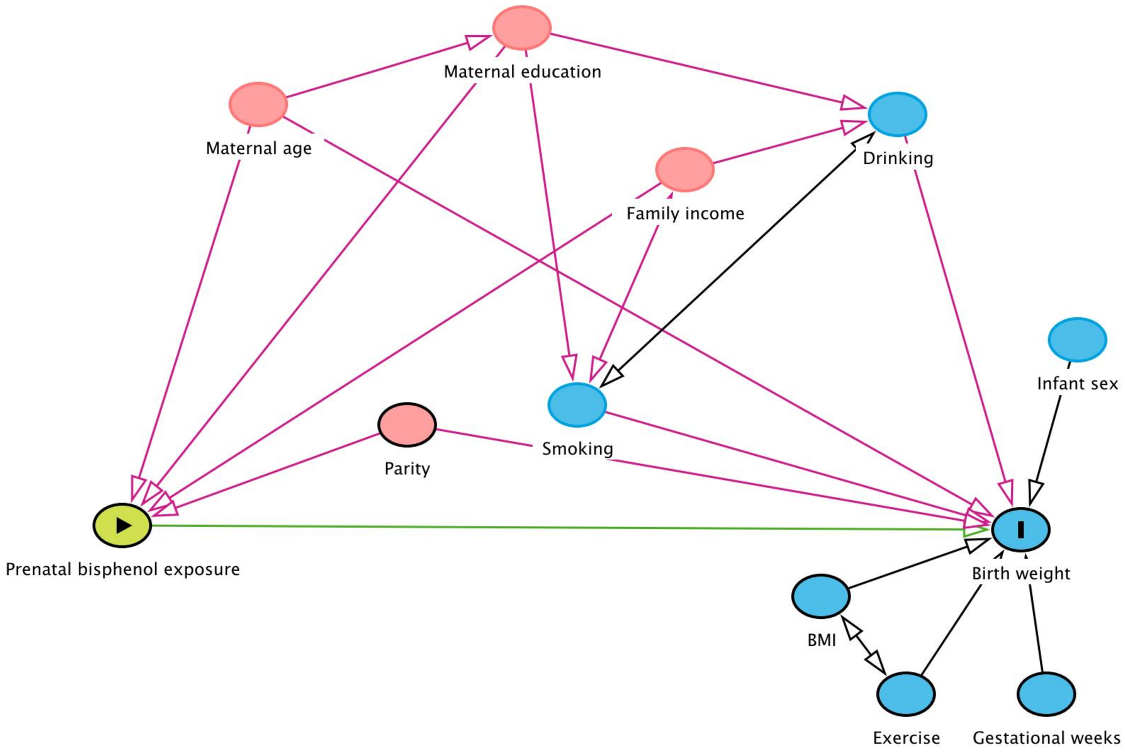

Figure S1. Directed acyclic graph (DAG).

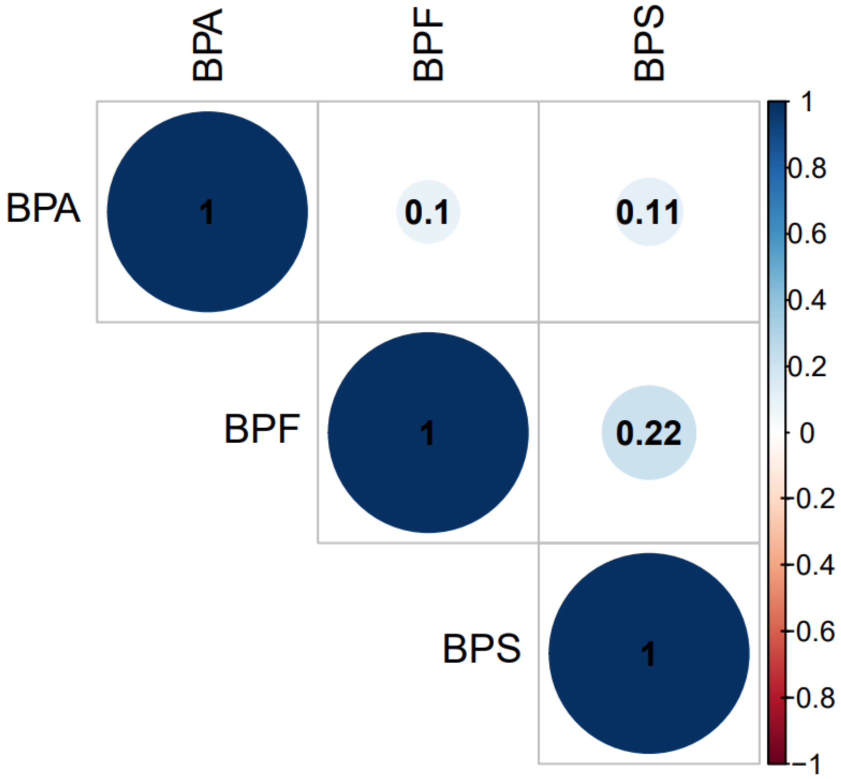

Figure S2. Correlation of BPA, BPF, and BPS.

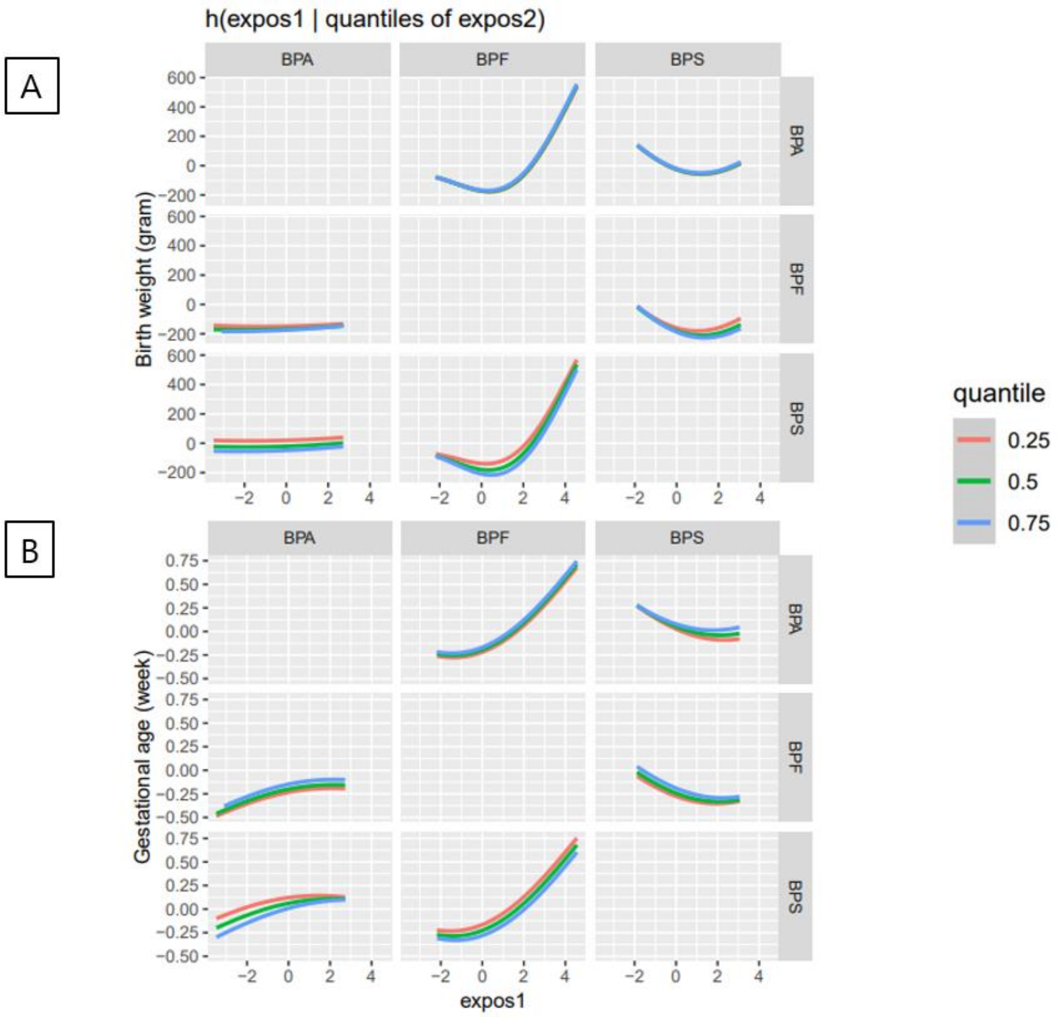

Figure S3. Bivariate exposure-response functions for one bisphenol (expos1) at quantiles of another bisphenol (expos2) on the (a) birth weight and (b) gestational age. Models were adjusted for maternal age, education, family income, smoking status, drinking status, body mass index, exercise, parity, infant sex, and gestational weeks (only in the case of birth weight).
